# Seroprevalence of Chagas Infection in the Donor Population

**DOI:** 10.1371/journal.pntd.0001771

**Published:** 2012-07-31

**Authors:** Ben A. Zaniello, Deborah A. Kessler, Katherine M. Vine, Kathleen M. Grima, Scott A. Weisenberg

**Affiliations:** 1 Department of Medicine, University of Washington, Seattle, Washington, United States of America; 2 Special Donor and Community Health Services, New York Blood Center, New York, New York, United States of America; 3 Special Patient Services, New York Blood Center, New York, New York, United States of America; 4 American Red Cross, Philadelphia, Pennsylvania, United States of America; 5 Alta Bates Summit Medical Center, Oakland, California, United States of America; National Institutes of Health, United States of America

## Abstract

We retrospectively calculated the prevalence and epidemiologic characteristics of Chagas infection in the New York blood donor population over three years utilizing the New York Blood Center's database of the New York metropolitan area donor population. Seventy *Trypanosoma cruzi* positive donors were identified from among 876,614 donors over a 3-year period, giving an adjusted prevalence of 0.0083%, with 0.0080% in 2007, 0.0073% in 2008, and 0.0097% in 2009. When filtered only for self-described “Hispanic/Latino” donors, there were 52 Chagas positive donors in that 3-year period (among 105,122 self-described Hispanic donors) with an adjusted prevalence of 0.052%, with 0.055% in 2007, 0.047% in 2008, and 0.053% in 2009. In conclusion, we found a persistent population of patients with Chagas infection in the New York metropolitan area donor population. There was geographic localization of cases which aligned with Latin American immigration clusters.

## Author Summary

Chagas Disease is a common and economically devastating disease of Latin America, with millions infected and many more at risk of infection. The hallmark of Chagas Disease is a long asymptomatic latent period (after an often tiny bug bite) followed by potentially fatal cardiac or gastrointestinal sequelae. Despite the significant number of immigrants from Chagas-endemic regions, prevalence data outside of its countries of origin remains limited. Our study looks at *Trypanosoma cruzi* infection in one group, blood donors in the New York metropolitan area, as this was a non invasive way to sample a sometimes difficult-to-reach population. We found that Chagas infection is in fact present, particularly in the Hispanic donors, at a consistent level over the three years we studied. We then compared the blood donor locations to a map of foreign born Hispanics in eastern Long Island in New York and found overlapping concentrations. This may mean that there is an opportunity for large scale community-focused screenings of foreign-born populations that could be both lifesaving and cost effective.

## Introduction

Chagas Disease is a common and economically devastating disease of Latin America, with an estimated infected population of over 7 million and over 100 million at risk [1]. Despite the significant number of immigrants from Chagas-endemic regions, prevalence data outside of its countries of origin remains limited [2]–[5]. Estimates of prevalence in non native areas are challenging given the asymptomatic nature of chronic Chagas Disease, the lack of familiarity of local physicians with this imported disease [6], and the often undocumented immigration status of some infected patients.

As a result, no large scale seroprevalence studies of immigrant populations have been done. Instead, many studies have followed a model first seen in Chagas endemic populations where the seroprevalence of Chagas infection in blood donors was used as proxy for overall population prevalence. However, donor seroprevalence of Chagas infection has been reported only from a limited set of populations, and epidemiologic associations of the donors are often lacking. We therefore retrospectively calculated Chagas infection seroprevalence and individual epidemiologic characteristics of infected patients in the greater New York blood donor population.

## Methods

The study was approved by the Institutional Review Board of both the New York Blood Center and Weill Cornell Medical Center. All data were analyzed anonymously. The New York Blood Center's database of the New York metropolitan area donor population was used to calculate the prevalence of Chagas infection in the general donor population. Chagas positivity was defined as a positive enzyme-linked immunosorbent assay (ELISA) screen (using the *T. cruzi* test system from Ortho Clinical Diagnostics in Raritan, NJ) with subsequent radioimmunoprecipitation assay (RIPA) confirmation (from Quest Diagnostics in Madison, NJ). The data set covered April 2007 to March 2010. Screening started in April 2007 so 2007 was adjusted to match the March to March 12 month period of other years by assuming the average monthly number of Chagas positive cases in 2007 continued for one more month. Collected variables included Sex, Racial/Ethnic Background, and Home Zip code, which were originally collected on the Blood Center's standard intake questionnaire given to individual donors.

## Results

**Figure 1:**
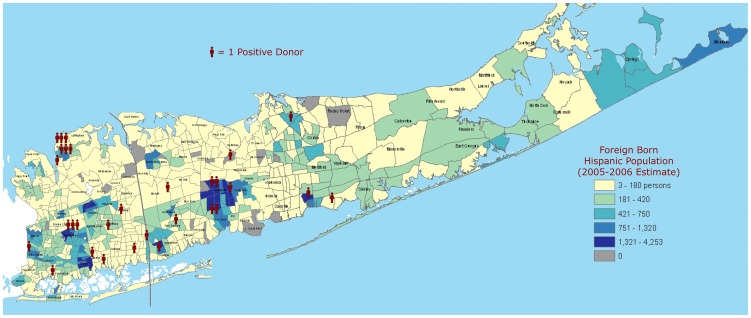
Chagas Positive Donors in Nassau and Suffolk Counties, New York, 2007 to 2009. Utilizing their contact zip codes, each donor was located on a map of eastern Long Island (New York) showing its Foreign Born Hispanic Population (population estimate from 2005–2006). Population analysis and underlying cartography modified/used with permission by Lee Hachadoorian, Center for Urban Research, City University of New York, 2007.

Seventy *Trypanosoma cruzi* positive unique donors were identified from among 876,614 donors over a 3 year period, giving an adjusted prevalence of 0.0083%, with 0.0080% in 2007, 0.0073% in 2008, and 0.0097% in 2009. When filtered only for self-described “Hispanic/Latino” donors, there were 52 Chagas positive donors in that 3 year period (from a sample of 105,122 self-described Hispanic donors) with an adjusted prevalence of 0.052%, with 0.055% in 2007, 0.047% in 2008, and 0.053% in 2009. The remaining 18 Chagas positive donors described themselves as either “Black” (1) or selected no Racial/Ethnic Background (17). Age range was 17 to 76 (median 43) and there were slightly more Females (54%). When mapped by zip code, the Chagas positive donor contact addresses showed a geographical concentration in one New York metropolitan area, with one notable city in that area seeing a cluster of Chagas positivity. Figure 1 shows one such concentration in Eastern Long Island, mapped on to 2000 Census data.

## Discussion

We found a persistent and possibly increasing population of patients with Chagas infection in the New York City Blood Donor population. Intriguingly, Chagas positivity appears to cluster in a limited set of geographic locations of that population.

This study expands what was previously known about Chagas prevalence outside its endemic regions, particularly in the United States. Previous studies have described the prevalence of Chagas infection in the donor population of Spain (0.62%) [4], Mexico (0.75%) [7], citing two examples, but the only detailed published U.S. data is from a sample set from 1994–1998, showing a 0.19% prevalence in Los Angeles and 0.08% in Miami [5]. The CDC has published more recent data in 2007 but with no detailed description of donor characteristics [8].

We also found geographic clustering of the donor population in areas with high Foreign Born Hispanic immigrant populations. For example, Eastern Long Island is unique in its large (50 k+) population of native Salvadorans [9], which may be mirrored by the geographic clustering of the positive donors in that area (please see Figure 1). Future efforts at identification of *Trypanosoma cruzi* infected populations may benefit from this donor-population derived “map” of areas of probable increased population Chagas prevalence. This has already been seen in Europe, where two studies, one In Spain and other in Switzerland, targeted high risk immigrant populations with direct screening (not during blood donation) and found a much higher seroprevalence than previously expected. They both confirmed, for example, that the Bolivian immigrant population is at particularly high risk for Chagas infection and merits focused outreach [10]–[11]. Additionally, while neither study looked at the economics of such screening, other studies indicate that even broader screening may make economic sense [12].

This study has several limitations. The prevalence in the Hispanic/Latino group may be underestimated due to lack of race self-identification among many donors, as 24% of Chagas positive donors did not indicate race and therefore could not be included in the “Hispanic/Latino” only results despite most studies indicating there are very few non-Hispanics with Chagas. Thus, the Hispanic/Latino prevalence could be as high as 0.067% over all three years if all the Chagas positive patients were in fact Latino. In addition, the donor's country of origin was not included in the questionnaire, and the Hispanic/Latino population in the study database was not segregated by place of birth. The Hispanic/Latino population in New York City includes Dominicans and Puerto Ricans (the largest Foreign born and the largest non Foreign born Hispanic groups in New York City, respectively [9]), groups not at high risk of Chagas positivity. Otherwise our data may have better mirrored the overall trend of increasing positivity, as seen in earlier, larger studies [4]. This increase would be consistent with the rise in immigration in the last decade of particular populations (i.e. rural Mexicans) with known higher Chagas positivity [13]. Also of note, blood donor populations do not necessarily mirror society as a whole [14]. However, this has been an accepted practice even in areas of highest Chagas seroprevalence given the difficulty of getting blood samples for the population most likely to be exposed to *T. cruzi* [15]. Finally it is important to note that no clinical follow up was available (Blood Center protocol is limited to referring them to an infectious disease physician), and thus we were unable to ascertain if any of the seropositive Donors were symptomatic.

These results indicate further analysis and outreach is warranted. Chagas Disease is an infection with both asymptomatic latency and debilitating sequelae in a substantial minority of infected patients. Identification, monitoring, and possible treatment of infected persons are best done through targeted identification and testing of at risk population groups. Diagnosis of Chagas infection in blood donors captures only a segment of the population infected with imported Chagas Disease. Characterization of high prevalence communities through blood donor seroprevalence suggests that follow up larger scale community-focused screenings of foreign-born populations could be both lifesaving and cost effective.
